# The Role of Synthesis Methods of Ceria-Based Catalysts in Soot Combustion

**DOI:** 10.3390/molecules30020358

**Published:** 2025-01-17

**Authors:** Gabriela Grzybek, Andrzej Wójtowicz, Piotr Legutko, Magdalena Greluk, Grzegorz Słowik, Andrzej Sienkiewicz, Andrzej Adamski, Andrzej Kotarba

**Affiliations:** 1 Faculty of Chemistry, Jagiellonian University in Krakow, Gronostajowa 2, 30-387 Krakow, Poland; andrzej.wojtowicz@student.uj.edu.pl (A.W.); piotr.legutko@uj.edu.pl (P.L.); a.adamski@uj.edu.pl (A.A.); kotarba@chemia.uj.edu.pl (A.K.); 2 Faculty of Chemistry, Maria Curie-Sklodowska University, Maria Curie-Sklodowska Sq. 3, 20-031 Lublin, Poland; grzegorz.slowik@mail.umcs.pl (G.S.); andrzej.sienkiewicz@mail.umcs.pl (A.S.)

**Keywords:** ceria, catalytic soot combustion, UV/Vis-DR studies, band gap, crystallite size

## Abstract

The removal of soot particles via high-performance catalysts is a critical area of research due to the growing concern regarding air pollution. Among various potential catalysts suitable for soot oxidation, cerium oxide-based materials have shown considerable promise. In this study, CeO_2_ samples obtained using a range of preparation methods (including hydrothermal synthesis (HT), sonochemical synthesis (SC), and hard template synthesis (TS)) were tested in soot combustion. They were compared to commercially available material (COM). All synthesized ceria catalysts were thoroughly characterized using XRD, RS, UV/Vis-DR, XPS, H_2_-TPR, SEM, and TEM techniques. As confirmed in the current study, every tested ceria sample can be used as an effective soot oxidation catalyst, with a temperature of 50% soot conversion not exceeding 400 °C in a tight contact mode. A strong correlation was observed between the catalysts’ Ce^3+^ concentration and activity, with higher Ce^3+^ levels leading to improved performance. These findings underscore the importance of synthesis in optimizing ceria-based catalysts for environmental applications.

## 1. Introduction

Anthropogenic soot emissions and their subsequent respiration by humans pose a significant threat to our health. Respirable ultrafine particles generated during the combustion of fossil fuels emitted from both mobile and stationary sources enter the atmosphere, becoming part of particulate matter (PM), which can enter the human body through the respiratory system and cause many serious health issues [1,2]. Additionally, as a greenhouse pollutant, soot also plays a negative role in climate change [3].

In 2021, the World Health Organization established strict limits on PM concentrations in its new air quality guidelines [4]. These highlighted the need to develop effective catalysts for low-temperature oxidation of soot accumulated on diesel particulate filters (DPFs).

The catalytic activity of materials in soot oxidation is assessed in two modes: a tight contact, where soot and the catalyst are thoroughly mixed (e.g., in a mortar or ball mill), and a loose contact, where materials are combined less intensively (e.g., by mixing with a spatula). Tight contact allows for the evaluation of the intrinsic activity of the catalyst, while loose contact better simulates real conditions in DPFs, where triphasic interactions (soot, catalyst, and oxidizing gas) are involved. In both cases, correlations have been observed between catalytic activity and material properties, such as grain morphology, surface area of a catalyst, exposed crystallographic surfaces, and the presence of lattice defects [5,6,7].

Ceria (cerium dioxide) has been identified as a promising catalyst for soot oxidation [5,8,9,10]. Its advantages include the ability to release oxygen from the crystal lattice of CeO_2_ at elevated temperatures, accompanied by changes in the oxidation state of cerium, which is able to enhance oxygen storage capacity, depending on crystallite size and temperature. Previous studies have shown that crystallographic surfaces with under-coordinated oxygen sites (stabilized on 100 and 110 crystallographic planes) exhibit higher catalytic activity than stable surfaces such as (111). Consequently, nanocubes with exposed (100) and (110) surfaces exhibit higher activity at low temperatures than polycrystalline octahedral CeO_2_ morphology with (111) surfaces exposed [11]. Moreover, a higher surface area and smaller crystallite size improve low-temperature activity due to a higher surface-to-volume ratio and the associated quantum effects [12,13,14].

Catalysts with more contact points towards the deposited soot also enhance catalytic performance, particularly in loose contact mode. For instance, ceria nanofibers were found to be more effective in comparison to materials with undefined morphologies [15,16]. Even better results were obtained in the case of hydrothermally synthesized CeO_2_ crystallizing in the form of micrometric star-like structures or nanocubes, which demonstrated lower soot oxidation temperatures [17].

Calcination temperature also impacts catalytic activity. Hydrothermally synthesized micrometric star-like structures calcined at 600 °C showed a reduced activity due to crystallite sintering, but their favorable morphology was preserved [18]. Conversely, calcination of CeO_2_ at temperatures above 500 °C promoted the exposure of catalytically active (100) and (110) surfaces, although it led to a decrease in their surface areas and the overall catalytic activities [11]. Moreover, various morphologies result in different lattice strains that can also control, to some extent, the activity of CeO_2_-based catalysts in soot combustion [19]. While various dependencies have been described in the literature [20,21,22], systematic comparisons of CeO_2_ of different morphologies prepared through diverse synthesis methods remain quite limited.

In this study, we investigated the soot combustion performance of three CeO_2_ materials synthesized using different methods, including hydrothermal synthesis (HT), sonochemical synthesis (SC), and hard template synthesis (TS). The goal was to determine and understand the functional dependence of the catalytic performance of the studied ceria-based samples on their synthetic origin. We examined all prepared materials regarding their composition, morphology, and surface properties using X-ray diffraction, spectroscopic and microscopic techniques, and temperature-programmed reduction studies. We compared their activity in the soot combustion to the activity of a commercial material (CeO_2_ (COM)) and discussed the observed differences in terms of the ceria morphology and cerium oxidation state.

## 2. Results and Discussion

### 2.1. Characterization of the Synthesized Materials

**Figure 1:**
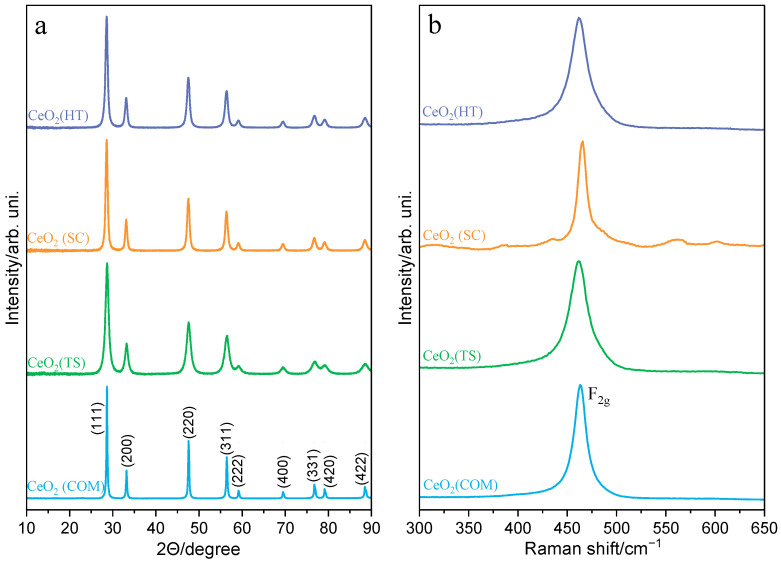
XRD powder diffraction patterns (**a**) and Raman spectra (**b**) of CeO_2_(HT), CeO_2_(SC), CeO_2_(TS), and CeO_2_(COM) samples.

The series of ceria catalysts were characterized in terms of their structural and surface properties, including chemical and phase compositions, reducibility, and morphology. The XRD patterns of the synthesized and reference commercially available ceria materials, presented in Figure 1a, proved that all materials crystallized with a typical structure with a cubic unit cell (Fm3m). The X-ray diffraction lines characteristic of the cubic ceria oxide phase (ICSD-28753) at 2θ equal to 28.6°, 33.2°, 47.6°, 56.4°, 59.2°, 69.5°, 76.8°, 79.1°, and 88.5° were assigned to (111), (200), (220), (311), (222), (400), (311), (420), and (422) planes, respectively. Compared to commercially available ceria, the synthesized samples display distinctly broader diffraction peaks, indicating the formation of smaller crystallites (Table 1). Complementary structural characterization was performed using Raman spectroscopy (Figure 1b). The Raman spectra of all ceria samples are dominated by one characteristic F_2g_ phonon mode, positioned around 462 cm^−1^, confirming a cubic fluorite CeO_2_ structure. According to the literature [23,24], this peak can shift to lower frequencies and broaden with decreased crystallite sizes. However, such relationships were not observed in the case of our CeO_2_ samples. Moreover, the Raman spectrum recorded for the CeO_2_(SC) sample shows very weak peaks between 525 and 575 cm^−1^. It is likely that they can be associated with oxygen defects occurring in the ceria lattice. The nature of these defects can be related to the presence of Frenkel-type defects formed by the displacement of O^2−^ ions from their lattice positions to the vicinal interstitial positions [25,26,27]. This effect can be induced by the partial reduction of CeO_2_ samples during their synthesis or subsequent thermal treatment.

**Figure 2:**
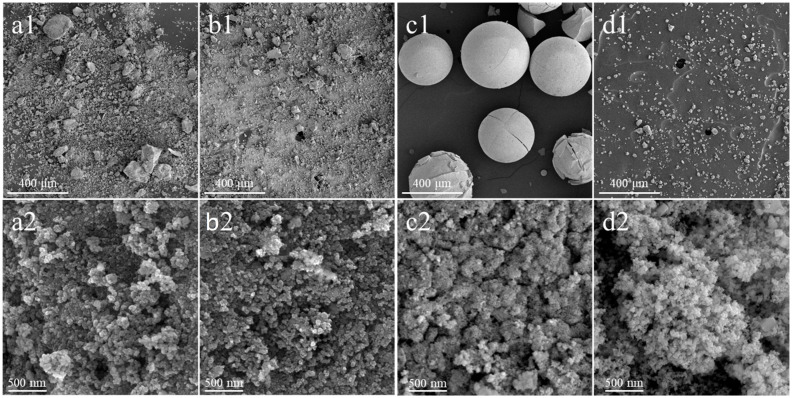
SEM micrographs recorded at the magnification of 100× (1) and 50,000× (2) of CeO_2_(HT) (**a1**,**a2**), CeO_2_(SC) (**b1**,**b2**), CeO_2_(TS) (**c1**,**c2**), and CeO_2_(COM) (**d1**,**d2**) samples.

Figure 2 presents a comparison of the surface morphology of the synthesized and commercial ceria materials carried out via scanning electron microscopy (SEM).

Among the samples studied, the CeO_2_(TS) sample, synthesized using the one-step hard template method, is quite characteristic in terms of morphology. As shown in Figure 2(c1), this material shows spherical particles, with cracks on the surface of the visible spheres formed due to the removal of volatile products formed during high-temperature synthesis [28]. In turn, the morphologies of the CeO_2_(HT), CeO_2_(SC), and materials are similar to those that are characteristic of commercial CeO_2_(COM). Irregular agglomerates with a broad size distribution induced by the presence of aggregated lamellar crystallites with irregular sizes and shapes are typical of such morphology.

**Figure 3:**
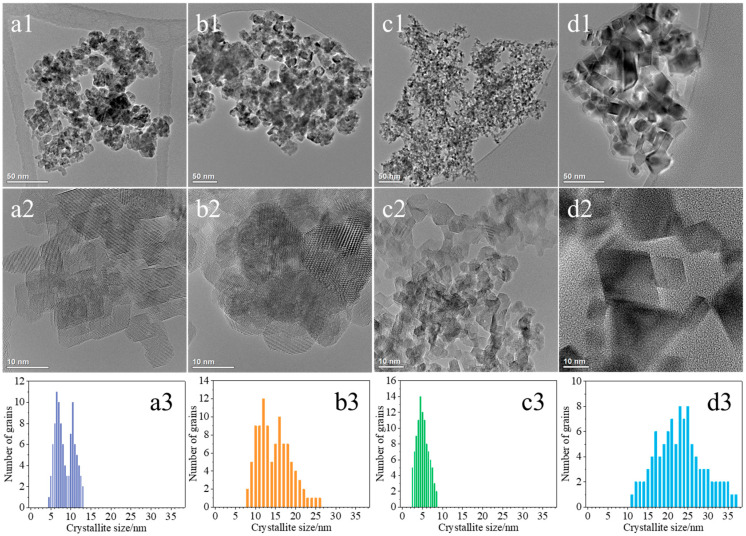
TEM images together with histograms of crystallite size distribution of CeO_2_(HT) (**a1**–**a3**), CeO_2_(SC) (**b1**–**b3**), CeO_2_(TS) (**c1**–**c3**), and CeO_2_(COM) (**d1**–**d3**) samples.

Figure 3 shows a detailed microscopic analysis of the ceria materials conducted using a transmission electron microscope (TEM). The crystallites in the examined samples exhibited diverse shapes and sizes depending on their preparative histories. In the case of the CeO_2_(HT) catalyst, the crystallites show irregular, sometimes elongated shapes and are frequently observed as agglomerates composed of large and small crystallites (Figure 3(a1,a2)). The CeO_2_(HT) sample is dominated by CeO_2_ crystallites belonging to two size ranges, i.e., 6–7 nm and 10–11 nm, with an average size of 8.4 nm (Figure 3(a3)). The crystallites of the CeO_2_(SC) sample displayed better uniformity in shape compared to the previous sample (Figure 3(b1,b2)). Most of the crystallites visible in the recorded micrographs exhibited round and regular shapes. Significant variability in crystallite size typical of this sample is also evident in the size distribution histogram, which distinctly shows two size maxima in the ranges of 10–13 nm and 15–18 nm with an average crystallite size of 14.3 nm (Figure 3(b3)).

The crystallites of the CeO_2_(TS) catalyst exhibit fairly regular and uniform shapes, with irregularities appearing less frequently (Figure 3(c1,c2)). The predominant ones were round and rectangular crystallites, and the elongated ones were observed only occasionally. The crystallites were generally small, not exceeding 10 nm, and the boundaries between them were quite visible. Nevertheless, the formation of agglomerates composed of several crystallites could still be observed. In the case of the CeO_2_(TS) sample, the average crystallite size is equal to 5.2 nm (Figure 3(c3)) and is the smallest among all the studied samples. Figure 3(d1,d2) show the crystallites of the commercial CeO_2_(COM) sample with highly diverse shapes and sizes. They exhibited relatively regular and repetitive shapes, including squares, rectangles, triangles, and rhombuses, and, less frequently, circular or elliptical shapes. The boundaries between individual crystallites were very well defined. Formation of agglomerates was rather uncommon. The average size of CeO_2_ crystallites typical of this sample reached approximately 22 nm (Figure 3(d3)), i.e., the largest among all the studied samples. Together with the average crystallite sizes determined from TEM images, the average diameters of crystallites (D_XRD_), calculated based on the Scherrer equation using a characteristic Bragg maximum at 2θ equal to 28.6° (111), are summarized in Table 1 for all investigated ceria-based samples. In most of the analyzed cases, the sizes observed through TEM are nearly half of those determined by the Scherrer method. The discrepancies between average sizes determined from XRD patterns using the Scherrer method and those obtained from TEM images can be attributed to the different physical principles and, hence, different information provided by these methods, exploiting individual particle sizes or average crystallite sizes, respectively. Thus, TEM provides localized, site-specific information, whereas XRD yields more average data, representing the entire sample analyzed.

**Figure 4:**
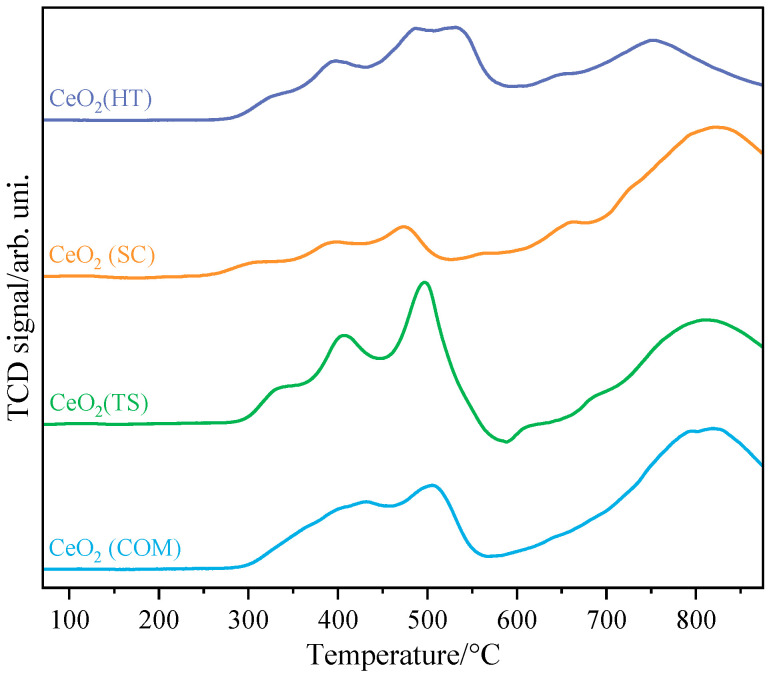
H_2_-TPR profiles collected for CeO_2_(HT), CeO_2_(SC), CeO_2_(TS), and CeO_2_(COM) samples.

The results of temperature-programmed reduction by hydrogen of the investigated CeO_2_ samples are depicted in Figure 4. Two main regions can be visible in the obtained thermograms; the first one, in the temperature window of 250–500 °C, can be attributed to the reduction in the surface of CeO_2_, while the second one, above 600 °C, is related to the reduction in bulk CeO_2_ [29,30,31].

Analysis of the first region leads us to the statement that ceria obtained using the sonochemical and hydrothermal methods exhibited the lowest threshold temperatures for reduction (around 250 °C), while the commercial sample, as well as the one obtained using hard template synthesis, started to be reduced at distinctly higher temperatures (around 300 °C). Moreover, the first group of samples exhibited more complex reduction behavior, which suggests more complex oxygen mobility dependent on sample preparation history [32].

**Figure 5:**
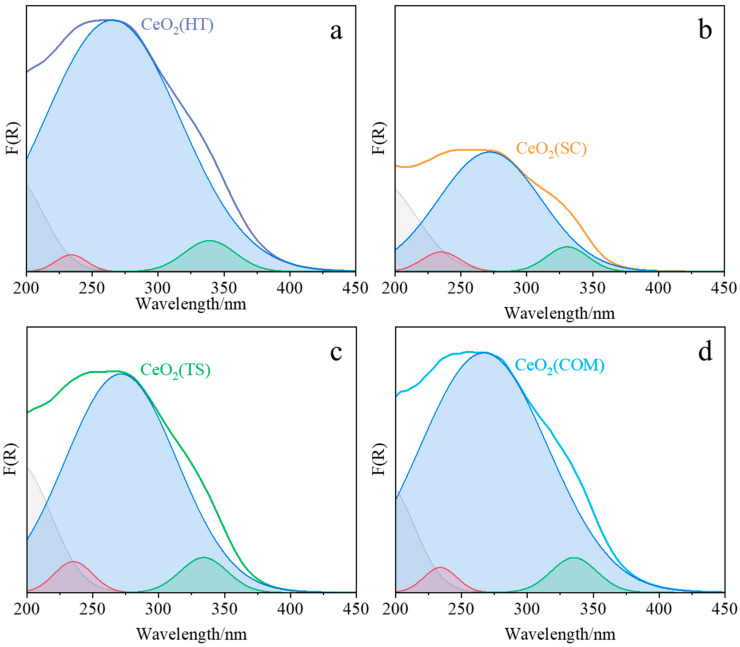
DR UV–Vis spectra of CeO_2_(HT) (**a**), CeO_2_(SC) (**b**), CeO_2_(TS) (**c**), and CeO_2_(COM) (**d**) samples deconvoluted into individual peaks.

The XRD, Raman, and H_2_-TPR characterization results were complemented by UV/Vis-DR spectroscopic studies (Figure 5 and Table 2). The observed maxima of the bands attributed to the ligand-to-metal charge transfer Ce^3+^ ← O^2−^ and Ce^4+^ ← O^2−^ transitions were determined in the ranges of 232–235 nm and 260–272 nm, respectively, which remains in agreement with the literature [33,34]. Similarly, the position of the observed interband transition, between 331 and 348 nm, agrees well with the literature data [33,34]. Based on the Tauc plots [35], the values of the band gap for each investigated ceria sample were estimated. The smallest value of the band gap was determined for CeO_2_(HT) and CeO_2_(COM) (both 3.48 eV), CeO_2_(TS) (3.52 eV), and CeO_2_ (SC) (3.53 eV).

**Figure 6:**
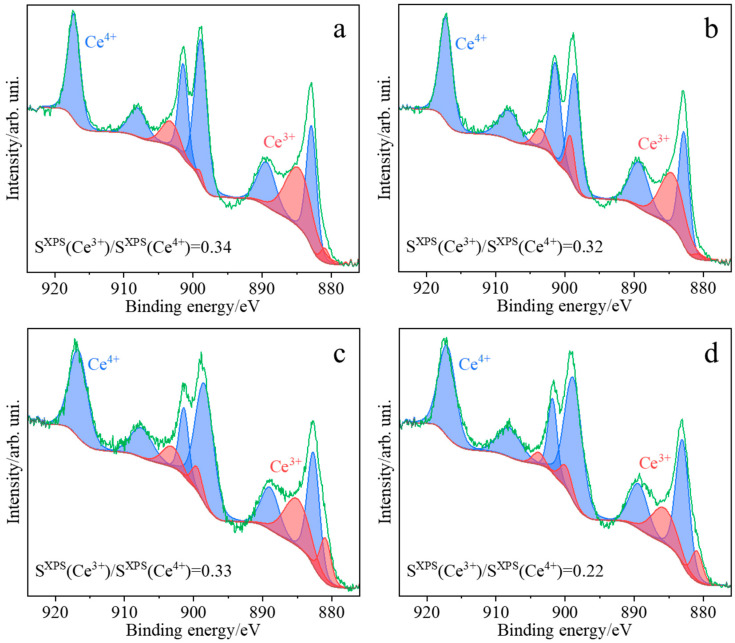
XPS high-resolution spectra collected in the Ce 3d window for the investigated samples CeO_2_(HT) (**a**), CeO_2_(SC) (**b**), CeO_2_(TS) (**c**), and CeO_2_(COM) (**d**).

Based on the deconvoluted UV/Vis-DR spectra, the decreasing order of Ce^3+^/Ce^4+^ concentration ratios for ceria samples obtained by various methods can be proposed as follows: CeO_2_(SC) > CeO_2_(TS) > CeO_2_(COM) > CeO_2_(HT) (Table 2). In parallel, the XPS spectra confirm that both oxidation states of cerium, Ce^4+^ and Ce^3+^, coexist on the surfaces of all the investigated samples (Figure 6). However, a comparison of the order of Ce^3+^/Ce^4+^ concentration ratios based on UV/Vis-DR (Table 2) with that obtained from XPS spectra (Table 3): CeO_2_(HT) > CeO_2_(TS) > CeO_2_(SC) > CeO_2_(COM) suggests that the composition of the surface was different from that determined for the bulk.

Ce^3+^/Ce^4+^ concentration ratios determined from deconvoluted XPS spectra distinctly differ for the samples synthesized via different preparation protocols. The samples obtained by hydrothermal synthesis (CeO_2_(HT)), hard template synthesis (CeO_2_(TS)), and sonochemical synthesis (CeO_2_(SC)) exhibited quite similar values of the Ce^3+^/Ce^4+^ ratio, at 0.34, 0.33, and 0.32, respectively, whereas the lowest value (0.22) was found for the commercial reference sample (CeO_2_(COM)). Based on the deconvolution of the O 1s region (Figure S1), it can be stated that the commercial reference sample exhibited the lowest ratio of the adsorbed oxygen to the lattice oxygen (0.18, see Table 3), while the remaining investigated samples can be ordered regarding their O_ads_/O_lattice_ as CeO_2_(HT) and CeO_2_(TS) (0.25), and the highest O_ads_/O_lattice_ ratio was found for CeO_2_(SC) (0.29). The ratio of total concentrations of cerium to oxygen is expected to be between 0.5 (for CeO_2_) and 0.66 (for Ce_2_O_3_). However, it should be noticed that the measured value of the Ce/O ratio is also affected by the presence of functional O-containing groups resulting from CO_2_ adsorption (carbon concentration varies from 20 to 50%) [36]. For most of the investigated samples, the determined Ce/O ratios (Table 3) are similar: 0.65 for CeO_2_(COM), 0.61 for CeO_2_(HT), and 0.59 for CeO_2_(SC). A significantly lower Ce/O ratio value was observed for the sample obtained using the hard template synthesis (0.18).

### 2.2. The Activity of the Ceria-Based Catalyst in the Soot Combustion Process

**Figure 7:**
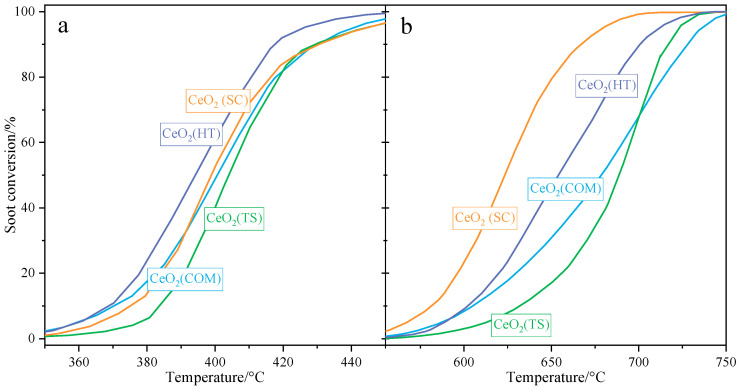
Results of catalytic tests in soot oxidation (TPO-QMS) over CeO_2_(HT), CeO_2_(SC), CeO_2_(TS), and CeO_2_(COM) samples performed in tight (**a**) and loose (**b**) contact, respectively.

Figure 7 below presents a comparison of the catalytic activities determined for ceria-based catalysts in both tight (a) and loose (b) contact modes. In tight contact mode, the CeO_2_(HT) catalyst exhibited the highest activity (*T*_50_ = 394 °C). Meanwhile, the remaining ceria samples showed similar, slightly lower activities (*T*_50_ ≈ 400 °C). In loose contact mode, the activity trend changed. For the CeO_2_(SC) sample, the highest catalytic performance was observed (T_5%_ = 623 °C), followed by that for CeO_2_(HT) (T_50_ = 654 °C). The activities determined for CeO_2_(TS) and for the commercial CeO_2_ samples were slightly lower, with T_50_ values of 689 °C and 679 °C, respectively. Based on the obtained results, it can be stated that all investigated samples showed a high degree of catalytic activities compared to the literature [10,37,38,39,40,41,42,43] and can be a promising premise for further optimization. Moreover, the catalysts studied showed almost 100% selectivity for CO_2_.

The active oxygen involved in soot combustion originates from both gaseous O_2_ and lattice O^2−^ within the catalyst. The activation of gaseous O_2_ is strongly influenced by oxygen vacancies, while the generation of active lattice O^2−^ depends on its mobility within the catalyst structure.

On ceria surfaces, gaseous O_2_ molecules accept electrons from Ce^3+^ ions associated with oxygen vacancies, forming active oxygen species such as O_2_^−^ and O^−^ (reaction 1). Consequently, soot can be oxidized by active oxygen species migrating from the catalyst’s surface (reaction 2). Also, the surface lattice O^2−^ can react with free carbon atoms in soot, reducing Ce^4+^ to Ce^3+^ (reaction 3). Additionally, highly mobile bulk lattice O^2−^ can migrate to the surface of ceria [18,44,45,46,47,48,49,50].

(1) $$\frac{x}{2}O_{2}+{\mathrm{Ce}}^{3+}\text{-}V_{O}\to {\mathrm{Ce}}^{4+}\text{-}O_{x}^{y-}$$

(2) $${\mathrm{Ce}}^{4+}\text{-}O_{x}^{y-}+\frac{x}{2}C\to \frac{x}{2}CO_{2}+{\mathrm{Ce}}^{3+}\text{-}V_{O}$$

(3) $$2{\mathrm{Ce}}^{4+}\text{-}O^{2-}\text{-}{\mathrm{Ce}}^{4+}+C\to 2{\mathrm{Ce}}^{3+}\text{-}V_{O}\text{-}{\mathrm{Ce}}^{3+}+CO_{2}$$

**Figure 8:**
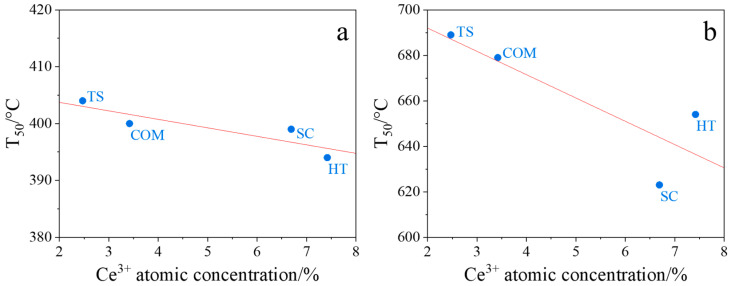
Correlations between the temperature of 50% soot conversion in tight (**a**) and loose (**b**) contact modes and the Ce^3+^ concentrations from XPS measurements for CeO_2_(HT), CeO_2_(SC), CeO_2_(TS), and CeO_2_(COM) samples.

Our studies in tight and loose contact modes revealed a correlation between the soot combustion performance of ceria oxides and the corresponding Ce^3+^ concentrations, determined from XPS spectra (Table 3). As can be seen in Figure 8, CeO_2_(HT) and CeO_2_(SC) samples, which exhibit the highest Ce^3+^ concentration, also showed the highest activity in soot combustion (the lowest T_50_ value, Table 4). Therefore, the most accepted mechanism for soot combustion over studied CeO_2_ samples relies on the reduction of O_2_ molecules during a catalytic redox reaction by the Ce^3+^ cations acting as a reservoir of electrons.

## 3. Materials and Methods

### 3.1. Catalyst Preparation

In this study, cerium oxide was prepared from a Ce(NO_3_)_3_∙6H_2_O (Merck, Darmstadt, Germany, 99.99%) precursor using various techniques, i.e., hydrothermal synthesis—CeO_2_(HT); sonochemical synthesis—CeO_2_(SC); thermal decomposition—CeO_2_(TD); and hard template synthesis—CeO_2_(TS). For comparison, commercially purchased cerium oxide—CeO_2_(COM) (Sigma-Aldrich, Saint Louis, MO, USA) was also examined.

#### 3.1.1. Hydrothermal Synthesis

For the CeO_2_(HT) sample synthesis, the Ce(NO_3_)_3_∙6H_2_O precursor (Merck, Darmstadt, Germany, 99.99%) was dissolved in distilled water. During vigorous mixing, the NaOH solution (3 M) was subsequently added to obtain a precipitate of cerium hydroxide, and stirring was continued for 30 min. Then, the suspension was transferred to a stainless-steel autoclave with a Teflon lining before being heated for 24 h at 150 °C. After cooling, the obtained yellow-white powder was centrifuged and washed three times with water and ethanol. The catalyst was dried overnight at 80 °C and finally calcinated for 4 h at 500 °C.

#### 3.1.2. Sonochemical Synthesis

For the CeO_2_(TD) sample synthesis, an ultrasonic probe with a diameter of 12 mm was used to sonicate the aqueous solution of Ce(NO_3_)_3_ (Merck, Darmstadt, Germany) for an hour (20 kHz, 500 W, amplitude 20%). Directly after starting the sonication, 3 mL of 25% ammonia solution was added dropwise to obtain a precipitate of cerium hydroxide. Subsequently, the suspension was centrifuged and dried overnight at 60 °C. Finally, the catalyst was calcinated for 4 h at 500 °C.

#### 3.1.3. Template Synthesis

The cerium oxide CeO_2_(TS) was produced according to the slightly modified process described in [27]. In brief, deionized water-washed, vacuum-dried, and sieved Amberlite XAD7HP beads (Supelco, Sigma-Aldrich, Saint Louis, MO, USA) with a diameter of >0.5 µm were swollen in an aqueous solution of 45% *w*/*w* cerium (III) nitrate hexahydrate (Aldrich, Darmstadt, Germany) at room temperature. The polymer absorbs approx. 3 times its dry mass and increases its volume. The swollen polymer beads were calcined for 4 h at 500 °C at a heating rate of 1 °C/min in an air atmosphere before being cooled to room temperature [23].

#### 3.1.4. Commercial Cerium Oxide

Commercial cerium oxide was purchased from Sigma-Aldrich (Saint Louis, MO, USA) (544841-5G).

### 3.2. Characterization of Materials

The phase composition was analyzed via X-ray diffraction (XRD) using a Rigaku Multiflex diffractometer with CuKα radiation (λ = 1.54 Å, 2θ: 5–90°, step: 0.02°, 2°/min) (Rigaku Corporation, Tokyo, Japan). The Raman spectra measurements were carried out using Renishaw InVia Qontor Raman Confocal Microscope (Renishaw, Wotton-under-Edge, Gloucestershire, UK). The samples were analyzed under magnification of 50 times. A laser with a wavelength of 785 nm and 0.5% of the total power was applied. The samples were exposed to laser radiation for 5 s. To obtain a desirable signal-to-noise ratio, 9 scans were accumulated. Each sample’s Raman spectra were collected at 5 different measurement points, and the Raman spectra obtained at different locations confirmed the samples’ homogeneity.

Catalyst imaging was carried out using a Quanta 3D FEG scanning electron microscope (SEM, FEI) (FEI Company, Hillsboro, OR, USA) with samples mounted on aluminum supported with carbon conductive tape and analyzed at 30 kV. Detailed morphological characterization was conducted using a Titan G2 scanning transmission electron microscope (TEM, FEI) (FEI Company, Hillsboro, OR, USA) at 300 kV. Element distribution was mapped via energy dispersion X-ray spectroscopy (EDX) in scanning TEM mode (STEM).

Spectra in the ultraviolet and visible regions in the diffuse reflectance mode (UV/Vis-DR) were collected by Lambda 650 (Perkin Elmer, Waltham, MA, USA) with the Praying Mantis (Harrick Scientific Products, New York, NY, USA) device. The experiments were performed in ambient conditions in the range of 200–900 nm, with a resolution of 1 nm. Spectra were converted according to the Kubelka–Munk equation and deconvoluted with Fityk software (version number: 0.8.0). A Tauc plot was used to determine the band gap.

Temperature-programmed reduction by hydrogen (H_2_-TPR) was performed in a quartz-tube reactor with a fixed bed. An amount of 50 mg of the sample was placed in the reactor and degassed in helium at 300 °C for 30 min. Then, the sample was cooled, and the gas mixture was changed to 5% H_2_ in Ar with a flow of 50 mL/min. Tests were performed for room temperature to 920 °C with a ramp of 5 °C/min. Changes in hydrogen concentration were monitored using a thermal conductivity detector (TCD, Agilent, Santa Clara, CA, USA).

X-ray photoelectron spectroscopy (XPS) studies were conducted using a PREVAC multi-chamber ultra-high vacuum system (PREVAC, Rogów, Poland) with a Scienta R4000 analyzer (Gammadata Scienta, Uppsala, Sweden). Spectra were collected using a Scienta SAX-100 X-ray source (Al Kα, 1486.6 eV) (Scienta Omicron AB, Uppsala, Sweden) and XM 650 Monochromator (Scienta Omicron AB, Uppsala, Sweden). Survey spectra were recorded at 200 eV pass energy (500 meV step) and high-resolution spectra (Ce 3d, O 1s, C 1s) at 50 eV pass energy (50–100 meV step). The base pressure was 5 × 10^−9^ mbar. Data analysis was performed with CasaXPS software (v2.3.23 PR1.0).

### 3.3. Catalytic Activity

The catalytic activity of the samples was measured in the reaction of model soot oxidation. An amount of 50 mg of a catalyst was mixed with 5 mg of soot (Printex U, Evonik Industries, Essen, Germany). In the case of tight contact mode, the mixture was ground in an agate mortar for 10 min; in the case of loose contact mode, a catalyst was shaken with soot in an Eppendorf probe for a minute. The prepared mixtures were placed in a quartz U-tube in a 5% oxygen flow in helium and heated at room temperature from a rate of 10 °C/min to 900 °C. The process of soot oxidation was monitored by probing the exhaust gases with a quadrupole mass spectrometer. The analyzed molecules included O_2_, CO_2_, CO, H_2_O, NO, and NO_2_. Because the observed low MS signal attributed to CO originates from CO_2_ fragmentation (according to Hiden Analytical, 11% of the CO_2_ signal corresponds in the MS spectra to CO, its fragmentation product), CO_2_ was thus regarded to be the only product of soot combustion.

## 4. Conclusions

A series of ceria materials were synthesized using various methods and analyzed in detail using XRD, Raman spectroscopy, UV-Vis, XPS, H_2_-TPR, SEM, and TEM techniques. Their catalytic behavior in soot combustion was investigated. Systematic research demonstrated that the catalytic activity of CeO_2_ in soot combustion can be effectively tailored using the appropriate method of synthesis. Hydrothermal synthesis (HT) and sonochemical synthesis (SC) produced CeO_2_ materials with more promising catalytic properties. All of them showed 50% soot conversions at temperatures below 400 °C in tight contact mode, demonstrating superior catalytic performance in comparison to the commercial CeO_2_ (COM) and CeO_2_(TS) samples. A clear correlation was established between Ce^3+^ concentration (as determined by XPS) and soot combustion performance. Higher Ce^3+^ concentrations were associated with enhanced catalytic activity. These findings highlight the importance of synthesis methods in optimizing ceria-based catalysts for environmental applications.

## Figures and Tables

**Table 1:** The average crystallite size of CeO_2_(HT), CeO_2_(SC), CeO_2_(TS), and CeO_2_(COM) samples calculated from XRD patterns using the Scherrer method and those determined from TEM images.

Sample	Crystallites Size—Scherrer Method (nm)	Average Crystallites Size—TEM (nm)
CeO_2_(HT)	16	8
CeO_2_(SC)	17	14
CeO_2_(TS)	11	5
CeO_2_(COM)	48	22

**Table 2:** Parameters obtained after deconvolution of the UV/Vis-DR spectra and Tauc plots fitted for CeO_2_(HT), CeO_2_(SC), CeO_2_(TS), and CeO_2_(COM) samples.

Sample	LM CT Ce^3+^ ← O^2−^ (nm)	LM CT Ce^4+^ ← O^2−^ (nm)	Interband (nm)	Ce (III): Ce (IV) Area Ratio	Band Gap (eV)
CeO_2_(HT)	234	265	339	0.0152	3.48
CeO_2_(SC)	235	272	331	0.0615	3.53
CeO_2_(TS)	235	271	334	0.0485	3.52
CeO_2_(COM)	234	267	335	0.0268	3.48

**Table 3:** Atomic concentrations of elements detected in CeO_2_(HT), CeO_2_(SC), CeO_2_(TS), and CeO_2_(COM) samples by XPS spectra.

Sample	Atomic Concentration (%)											
	Ce^(III)^	Ce^(IV)^	Ce (All)	O_latt_	O_ads_	O (All)	C_aliph_	C_carbox_	C (All)	Ce/O	Ce^(III)^/Ce^(IV)^	O_ads_/O_latt_
CeO_2_(HT)	7.42	21.82	29.24	38.91	9.8	48.71	11.16	10.9	22.06	0.60	0.34	0.25
CeO_2_(SC)	6.69	21.19	27.88	37.39	10.95	48.34	15.58	8.21	23.79	0.58	0.32	0.29
CeO_2_(TS)	2.47	7.44	9.91	41.79	10.25	52.04	31.31	6.73	38.04	0.19	0.33	0.25
CeO_2_(COM)	3.42	15.49	18.91	24.47	4.31	28.78	40.37	11.94	52.31	0.66	0.22	0.18

**Table 4:** The catalytic activity parameters determined for the investigated ceria samples tested in soot combustion.

Sample	Characteristic Temperatures of Soot Conversion/°C					
	Tight Contact Mode			Loose Contact Mode		
	*T*_10_	*T*_50_	*T*_90_	*T*_10_	*T*_50_	*T*_90_
CeO_2_(HT)	368	394	418	602	654	701
CeO_2_(SC)	375	400	431	581	623	668
CeO_2_(TS)	383	404	430	632	689	717
CeO_2_(COM)	370	402	430	605	679	728

## Data Availability

The data will be available upon request.

## Supplementary Materials

The following supporting information can be downloaded at https://www.mdpi.com/article/10.3390/molecules30020358/s1. Figure S1. XPS high-resolution spectra collected in O 1s window for the investigated samples: CeO_2_(HT) (a), CeO_2_(SC) (b), CeO_2_(TS) (c) and CeO_2_(COM) (d). Figure S2. Survey XPS spectra collected for the investigated samples: CeO_2_(HT) (a), CeO_2_(SC) (b), CeO_2_(TS) (c) and CeO_2_(COM) (d).
