# Supplementary material for: The Role of Synthesis Methods of Ceria-Based Catalysts in Soot Combustion
Source: Molecules. 2025 Jan 17;30(2):358. doi: 10.3390/molecules30020358 (PMC11767885; doi:10.3390/molecules30020358)
Supplement: Supplementary file 1 [file molecules-30-00358-s001.zip › molecules-3359423-supplementary.pdf]

The Role of Synthesis Methods of Ceria-Based Catalysts in Soot Combustion

Gabriela Grzybek [1,*], Andrzej Wójtowicz [1], Piotr Legutko [1], Magdalena Greluk [2,*], Grzegorz Słowik [2], Andrzej Sienkiewicz [2], Andrzej Adamski [1] and Andrzej Kotarba [1]

[1] Faculty of Chemistry, Jagiellonian University in Krakow, Gronostajowa 2, 30-387 Krakow, Poland; andrzej.wojtowicz@student.uj.edu.pl (A.W.); piotr.legutko@uj.edu.pl (P.L.); a.adamski@uj.edu.pl (A.A.); kotarba@chemia.uj.edu.pl (A.K.)

[2] Faculty of Chemistry, Maria Curie-Sklodowska University, Maria Curie-Sklodowska Sq. 3, 20-031 Lublin, Poland; grzegorz.slowik@mail.umcs.pl (G.S.); andrzej.sienkiewicz@mail.umcs.pl (A.S.)

* Correspondence: g.grzybek@uj.edu.pl (G.G.); magdalena.greluk@mail.umcs.pl (M.G.)

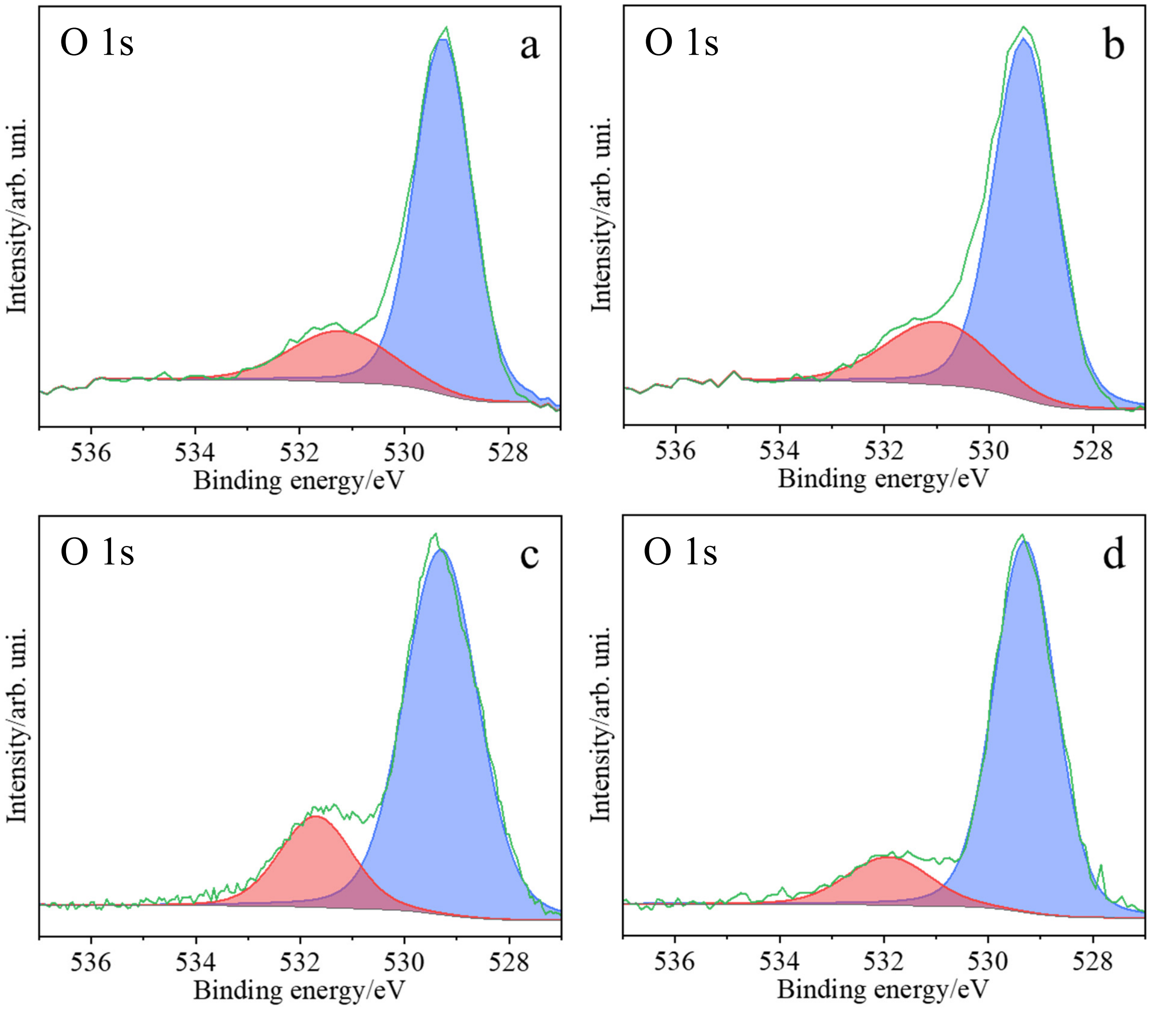

Figure S1. XPS high-resolution spectra collected in O 1s window for the investigated samples: CeO_2(HT) (a), CeO_2(SC) (b), CeO_2(TS) (c) and CeO_2(COM) (d).

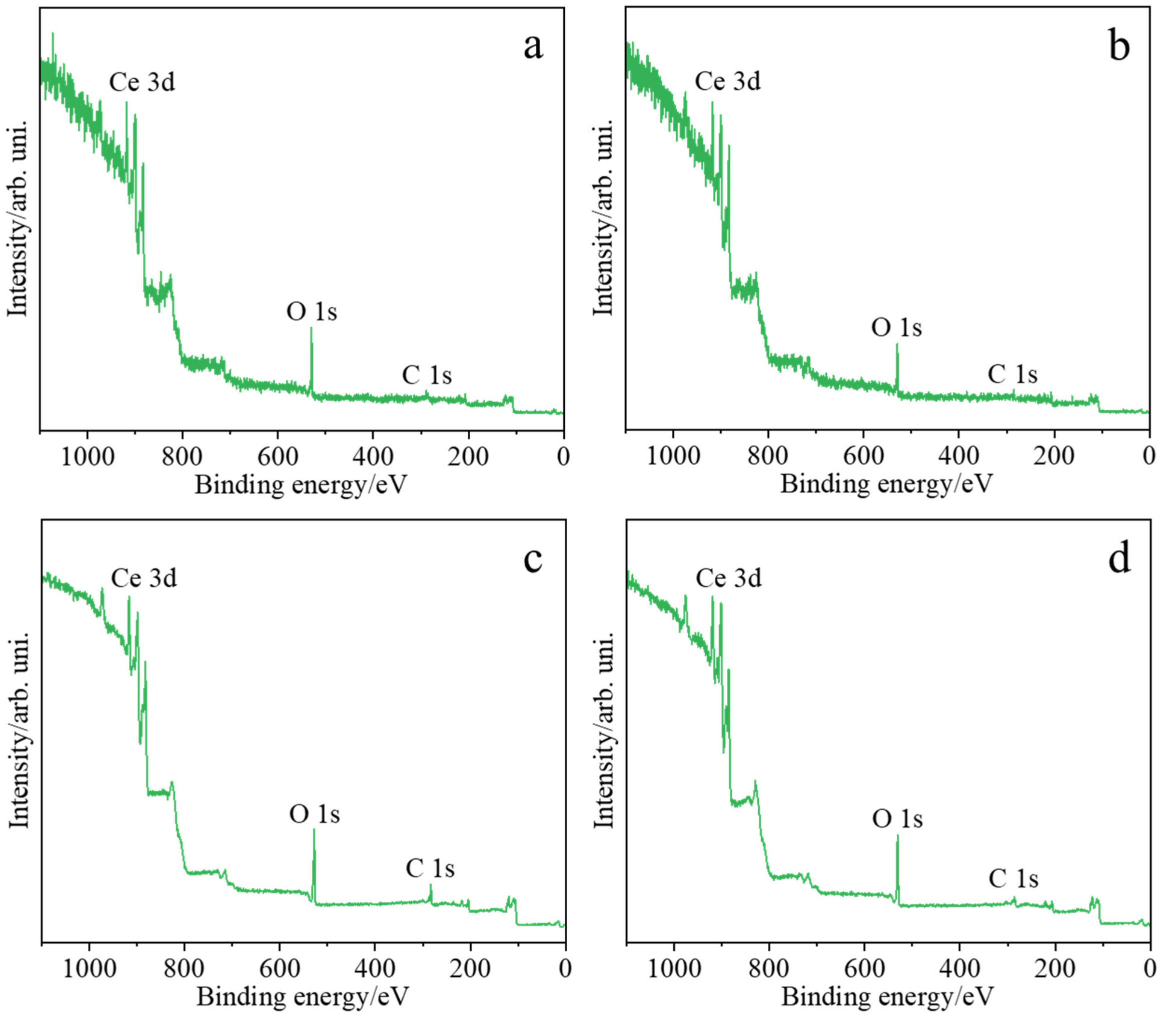

Figure S2. Survey XPS spectra collected for the investigated samples: CeO_2(HT) (a), CeO_2(SC) (b), CeO_2(TS) (c) and CeO_2(COM) (d).
